# The Effects of e-interventions on the Medical Outcomes of Hemodialysis Patients: A Retrospective Matched Patient Cohort Study

**DOI:** 10.1038/s41598-017-02815-9

**Published:** 2017-06-07

**Authors:** Chang-Chyi Jenq, Cheng-Chieh Hung, Kuo-Chang Juan, Kuang-Hung Hsu

**Affiliations:** 1 Department of Nephrology, Chang Gung Memorial Hospital, Linkou, Taiwan; 2 College of Medicine, Chang Gung University, Taoyuan City, Taiwan; 3 Nephrology Department, Everan Hospital, Taichung, Taiwan; 4 Laboratory for Epidemiology, Department of Health Care Management, Chang Gung University, Taoyuan City, Taiwan; 5 Healthy Aging Research Center, Chang Gung University, Taoyuan City, Taiwan; 6 Department of Urology, Chang Gung Memorial Hospital, Taoyuan City, Taiwan

## Abstract

Aggressively applying e-interventions in the health care industry has become a global trend to improve the quality of medical care. The present retrospective study evaluated the effect of electronic information systems on the quality of medical care provide to hemodialysis (HD) patients. In total, 600 patients (300 patients each in the e-intervention and non-e-intervention groups, were matched for sex, age, HD duration, diabetes, and hypertension) receiving HD at the study institute for four years were included in this study. The e-intervention group had significantly fewer hospitalization days than the non-e-intervention group. Cox regression analysis demonstrated that the non-e-intervention group had a significantly higher mortality rate than the e-intervention group. Stratified analysis revealed significant differences between the e-intervention and non-e-intervention groups in their serum albumin levels, urea reduction ratios, and cardiothoracic ratios at 1-year follow-up. The patients in the e-intervention group had a significantly higher HD blood flow rate, fewer hospitalization days and a lower 4-year all-cause mortality rate than those in the non-e-intervention group. The implementation of the e-intervention improved patient outcomes, but additional studies are required to evaluate the cost effectiveness of such implementations.

## Introduction

A large prospective cohort study by the Taiwan National Institutes of Health in 2008 reported that nearly 2.3 million people in Taiwan (12% of the population) have chronic kidney disease (CKD)^1^. When CKD progresses to end-stage renal disease (ESRD), patients require long-term dialysis to sustain life. The medical care system in Taiwan is a single-payer compulsory insurance program. The medical expenditures related to hemodialysis (HD) have increased because of an increase in the number of HD patients over the past few decades^2^. Therefore, monitoring the quality of HD and reducing the complication rate in HD patients have become important concerns that should be addressed to contain medical costs in health care systems.

HD requires vigorous quality control to ensure patient safety and provide high quality care^3^. Electronic information technology can provide potential benefits in health-care management^4^. Moreover, aggressive application electronic information technology in the health care industry, referred to as e-interventions, has become a global trend in recent years, and is considered as a necessary tool for providing high quality care^5^. The e-interventions has been managed the vast clinical data of HD patients^6, 7^, thus improving the quality of medical care^8, 9^.

The present study evaluated the effects of e-interventions on the quality of medical care, particularly on the 4-year all-cause mortality rate, among HD patients in the largest medical system in Taiwan.

## Results

### Demographic and clinical characteristics of the study groups and the final outcomes

The mean age of the 600 study patients was 59 years (25–87 years), with a similar male-female distribution (52% vs. 48%). More patients with an education level below junior high school (relatively low education level) were observed in the e-intervention group (74%) than in the non-e-intervention group (65.67%). More patients were married in the e-intervention group (72%) than in the non-e-intervention group (39.33%). Regarding HD vintage, the duration of HD in the e-intervention and non-e-intervention groups was 1–28 years. Chronic glomerulonephritis was the most frequent cause of ESRD in the non-e-intervention group (49.67%), and hypertension was the major cause of ESRD in the e-intervention group (37.67%). More patients had hepatitis B virus infections in the e-intervention group (17%) than in the non-e-intervention group (6.67%). Before the e-intervention, the two study groups differed significantly in their proportion of anuria, haematocrit levels, blood sugar levels, proportion of iron supply, monthly erythropoietin usage, and cardiothoracic ratio. Regarding the outcome variables, the e-intervention group had significantly fewer hospitalization days than the non-e-intervention group at 1-year post e-intervention (Table 1). The causes of death during the study period and the mortality rates are shown in Table 1. The e-intervention group had a relatively lower 4-year all-cause mortality rate than the non-e-intervention group.

**Table 1:** Demographic factors and clinical characteristics of the study patients.

Variables	All (n = 600) Frequency (%)/Mean ± SD	Non-e group (n = 300) Frequency (%)/Mean ± SD	e group (n =300) Frequency (%)/Mean ± SD	p value
**Basic demographics**				
**Age (years)**	58.96 ± 11	59.04 ± 11.15	58.88 ± 10.86	0.8641
**Sex**				
Male	312 (52.00)	156 (52.00)	156 (52.00)	1.0000
Female	288 (48.00)	144 (48.00)	144 (48.00)	
**Education level**				
Under junior high school	419 (69.83)	197 (65.67)	222 (74.00)	0.0328
Senior high school and above	181 (30.17)	103 (34.33)	78 (26.00)	
**Occupation**				
Laborer	65 (10.83)	34 (11.3)	31 (10.3)	0.9245
Non-laborer	111 (18.50)	55 (18.3)	56 (18.7)	
Unemployed	424 (70.67)	211 (70.3)	213 (71.0)	
**Marital status**				
Married	334 (55.67)	118 (39.33)	216 (72.00)	<0.0001
**Body mass index (kg/m** ^**2**^ **)**	22.56 ± 3.4	22.4 ± 3.55	22.86 ± 3.1	0.1808
**Medical characteristics and co-morbidities**				
**Duration of HD (years)**	7.19 ± 5.29	7.16 ± 5.31	7.22 ± 5.28	0.9019
**The cause of end-stage renal disease**				
CGN	258 (43.00)	149 (49.67)	109 (36.33)	<0.0001
Diabetes mellitus	123 (20.50)	60 (20.00)	63 (21.00)	
Hypertension	155 (25.83)	42 (14.00)	113 (37.67)	
Others	64 (10.67)	15 (16.33)	15 (5.00)	
**Co-morbidities**				
**HBV antigen**				
Positive	71 (11.83)	20 (6.67)	51 (17.00)	<0.0001
**HCV antibody**				
Positive	96 (16.00)	51 (17.00)	45 (15.00)	0.5039
**Diabetes mellitus**				
Yes	120 (20.00)	60 (20.00)	60 (20.00)	1.0000
**Hypertension**				
Yes	202 (33.67)	101 (33.67)	101 (33.67)	1.0000
**Vascular access type**				
A-V fistula	454 (75.67)	225 (75.00)	229 (76.33)	0.4248
A-V graft	121 (20.17)	65 (21.67)	56 (18.67)	
Double lumen catheter^	25 (4.17)	10 (3.33)	15 (5.00)	
**Physiological and biochemical variables before the e-intervention**				
Yes	542 (90.33)	260 (86.67)	282 (94.00)	0.0021
Surface area of the dialyzer (m^2^)	1.96 ± 0.29	1.98 ± 0.28	1.94 ± 0.3	0.1484
Blood flow (mL/min)	288.18 ± 40.04	285.67 ± 36.91	290.7 ± 42.86	0.1238
Albumin (g/dL)	3.95 ± 0.34	3.93 ± 0.34	3.97 ± 0.34	0.1892
Pre-HD creatinine (mg/dL)	10.64 ± 2.34	10.54 ± 2.35	10.74 ± 2.33	0.3254
nPCR (g/kg/day)	1.26 ± 0.38	1.25 ± 0.37	1.26 ± 0.39	0.9166
TACurea (mg/dL)	41.55 ± 10.92	41.21 ± 10.95	41.86 ± 10.9	0.4758
Potassium (meq/L)	4.92 ± 0.72	4.97 ± 0.71	4.88 ± 0.72	0.1469
Kt/V	1.79 ± 0.32	1.79 ± 0.35	1.8 ± 0.29	0.6175
URR	0.77 ± 0.06	0.77 ± 0.06	0.76 ± 0.07	0.1877
Hematocrit (Hct, %)	31.86 ± 4.09	32.2 ± 4.1	31.52 ±4.06	0.0436
Iron administration by vein				
Yes	124 (20.67)	42 (14.00)	82 (27.33)	<0.0001
Monthly EPO usage (1000U/month)*	16 (8, 22)	16 (8, 22)	18 (11, 22)	0.0090
Calcium (mg/dL)	9.67 ± 0.94	9.64 ± 0.93	9.71 ± 0.95	0.4001
Phosphate (mg/dL)	4.71 ± 1.37	4.63 ± 1.33	4.79 ± 1.41	0.1679
iPTH (ng/mL)*	134.15	138.3	127.3	0.6062
	(49.9, 312.15)	(49.9, 324.2)	(50, 288.3)	
Total cholesterol (mg/dL)	174.9 ± 37.59	172.3 ± 36.94	177.39 ± 38.1	0.1064
Glucose (mg/dL)*	97 (85, 132)	99.5 (87,133.5)	95 (82, 129)	0.0277
Cardiothoracic ratio	0.49 ± 0.06	0.5 ± 0.07	0.48 ± 0.06	<0.0001
**Outcome variables**				
Total length of hospitalization (days/person-year)	1.63 ± 8.99	3.26 ± 12.51	0 ± 0	<0.0001
**4-year all-cause mortality n(%)**				
Deaths	105 (17.50)	59 (19.67)	46 (15.33)	0.1620
Survivors	495 (82.50)	241 (80.33)	254 (84.67)	
Cause of death n (%)				
Gastro-entero-intestine (GI)	14 (10.37)	8 (10.81)	6 (9.84)	0.5890
Cardiovascular Diseases (CVDs)	37 (27.41)	18 (24.32)	19 (31.15)	
Infections	76 (56.30)	42 (56.76)	34 (55.74)	
Others (including injuries, lung diseases, etc)	8 (5.93)	6 (8.11)	2 (3.28)	

e group: e-intervention group; non-e group: non-e-intervention group; HD: hemodialysis; CGN: chronic glomerulonephritis; HBV: hepatitis B virus; HCV: hepatitis C virus; A-V: arteriovenous; nPCR: normalized protein catabolic rate; TACurea: time-averaged concentration of urea; URR: urea reduction ratio; EPO: erythropoietin; iPTH: intact parathyroid hormone.

^ Double lumen catheter includes tunneled cuffed catheter

*The variables with skew distribution were presented as median (1st-quartile, 3rd-quartile) and tested with Mann-Whitney U-test accordingly.

### Statistical analysis results for e-intervention effects

**Figure 1:**
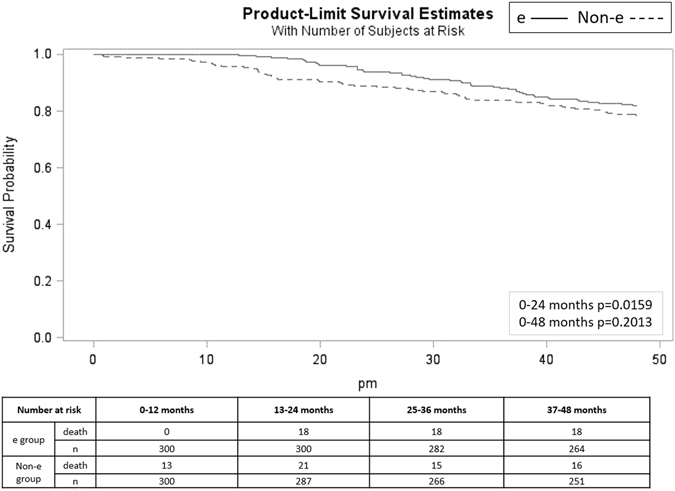
Survival functions Kaplan-Meier survival analysis in the 600 patients according to e-intervention or not showed four-year survival curve and number at risk table.

In multivariable Cox regression analysis, patients in the non-e-intervention group (hazard ratio [HR] = 1.991; 95% confidence interval [CI] = 1.194~3.317) were more likely to die within the 4-year follow-up period than those in the e-intervention group, after adjusting for other variables based on the model selection criteria. In addition, significantly higher mortality rates were observed in patients with a relatively lower education level, unmarried patients, those with lower pre-HD serum creatinine levels, and those with higher sugar levels (Table 2). Stratified analysis revealed significant differences in the serum albumin levels, urea reduction ratio (URR), cardiothoracic ratio and HD blood flow rate of the e-intervention and non-e-intervention groups at 1-year follow-up. The e-intervention group had higher serum albumin levels (3.97 vs. 3.90 g/dL, *p* = 0.0041), a higher URR (0.78 vs. 0.76, *p* < 0.0001), a lower cardiothoracic ratio (0.48 vs. 0.50, *p* < 0.0001), and a faster HD blood flow rate (291 vs. 284 mL/min, *p* = 0.0032) than the non-e-intervention group (Table 3). Kaplan-Meier survival curve analysis demonstrated that the e-intervention group had a significantly lower 2-year all-cause mortality rate than the non-e-intervention group (Figure 1).

**Table 2:** Factors associated with 4-year all-cause mortality of the hemodialysis patients.

Variables	Person-months	Number of death	Univariate measure		Multivariate-adjusted measure	
			HR	95%CI	HR	95%CI
**Study groups**						
Non-e-intervention	12880.8	65	1.264	(0.881, 1.813)	1.991*	(1.194, 3.317)
e-intervention	13442.5	54	—	—	—	—
**Basic demographics**						
**Education level**						
Under junior high school	17977.5	104	3.267*	(1.901, 5.615)	1.913*	(1.012, 3.616)
Senior high school and above	8345.8	15	—	—	—	—
**Occupation**						
Laborer	3076.0	4	—	—		
Non-laborer	5070.4	16	2.438	(0.815, 7.293)		
Unemployment	18177.0	99	4.274*	(1.573, 11.613)		
**Marital status**						
Married	13706.1	16	—	—	—	—
Not married	12617.2	103	6.125*	(3.616,10.375)	5.500*	(2.934, 10.311)
**Body mass index (each increment)**	—	—	0.981	(0.926, 1.039)		
**Disease characteristics and co-morbidities**						
**Duration of HD (years)**	—	—	0.973	(0.939, 1.009)		
**Co-morbidities**						
**HBV antigen**						
Negative	23076.8	109	—	—	—	—
Positive	3246.4	10	0.647	(0.339, 1.237)	1.246	(0.598, 2.598)
**HCV antibody**						
Negative	22017.7	97	—	—		
Positive	4305.5	22	1.153	(0.726, 1.832)		
**Diabetes mellitus**						
No	21570.8	70	—	—		
Yes	4752.5	49	3.271*	(2.269, 4.715)		
**Hypertension**						
No	17324.5	81	—	—		
Yes	8998.8	38	0.900	(0.612, 1.324)		
**Vascular access type**						
A-V fistula	20119.3	75	—	—	—	—
A-V graft	5252.0	31	1.596*	(1.050, 2.425)	1.589	(0.953, 2.649)
Double lumen catheter	952.0	13	3.822*	(2.119, 6.894)	1.975	(0.952, 4.095)
**Physiological and biochemical variables before the e-intervention**						
Daily urine amount (mL/day)	—	—	0.970*	(0.949, 0.992)		
Surface area of the dialyzer (m^2^)	—	—	1.316	(0.695, 2.491)		
Albumin (g/dL)	—	—	0.249*	(0.150, 0.414)	0.771	(0.356, 1.669)
Pre-HD creatinine (mg/dL)	—	—	0.748*	(0.686, 0.815)	0.813*	(0.708, 0.933)
nPCR (g/kg/day)	—	—	0.452*	(0.257, 0.794)		
TACurea (mg/dL)	—	—	0.963*	(0.946, 0.981)		
Potassium (meq/L)	—	—	0.647*	(0.497, 0.843)		
Kt/V	—	—	0.881	(0.499, 1.555)		
URR	—	—	2.572	(0.153, 43.341)	4.251	(0.132, 136.648)
Hematocrit (%)	—	—	0.963	(0.920, 1.008)	1.034	(0.956, 1.117)
Iron supply						
No	20830.5	91	—	—	—	—
Yes	5492.8	28	1.166	(0.763, 1.781)	1.274	(0.780, 2.081)
Monthly EPO usage (1000U/month)	—	—	1.016	(0.995, 1.037)	1.010	(0.981, 1.041)
Calcium (mg/dL)	—	—	0.856	(0.701, 1.047)		
Phosphate (mg/dL)	—	—	0.836*	(0.727, 0.961)	1.107	(0.918, 1.334)
iPTH (ng/mL)	—	—	0.999	(0.999, 1.000)		
Total cholesterol (mg/dL)	—	—	0.991*	(0.985, 0.996)	0.994	(0.987, 1)
Cardiothoracic ratio	—	—	555.934*	(41.821, 7390.177)	28.888	(0.818, 1020.780)
Glucose (mg/dL)	—	—	1.004*	(1.003, 1.006)	1.003*	(1.001, 1.005)
Blood flow (cc/min)	—	—	0.989*	(0.984, 0.993)	1.002	(0.994, 1.009)
Anuria						
No	2778.5	1	—	—	—	—
Yes	23544.8	118	14.197*	(1.984, 101.596)	6.031	(0.809, 44.966)
Hospitalization days	—	—	1.017*	(1.007, 1.027)		
**Cause of death**						
GI	11686.3	35	1.818	(0.944, 3.503)		
CVDs	4896.5	49	6.262*	(3.329, 11.779)		
Infections	7235.4	12	5.772*	(2.871, 11.602)		
Others	2505.1	23	—	—		

HD: hemodialysis; A-V: arteriovenous; EPO: erythropoietin; iPTH: intact parathyroid hormone; URR: urea reduction ratio; HBV: hepatitis B virus; HCV: hepatitis C virus; nPCR: normalized protein catabolic rate; TACurea: time-averaged concentration of urea

*p-value < 0.05 in the univariate and multivariate-adjusted analyses.

**Table 3:** Clinical characteristics and outcomes associated factors of the study patients one year after the e-intervention.

Variables	All (n = 600)		Non-e group (n = 300)		e group (n = 300)		p- value^
	Frequency(%)/Mean ± SD		Frequency(%)/Mean ± SD		Frequency(%)/Mean ± SD		
**Outcome variables**							
**Hospitalization days (days/person-years)**	1.66	±9.08	3.39	±12.77	0.00	±0.00	<0.0001
**Blood flow (cc/min)**	287.39	±39.31	283.52	±35.97	291.08	±41.99	0.0032
**1-year all-cause mortality**							
Deaths	13 (2.17)		13 (4.33)		0 (0.0)		<0.0001
Survivors	909 (97.5)		287 (95.67)		300 (100.0)		
**Physiological and biochemical variables**							
Albumin (g/dL)	3.94	±0.33	3.9	±0.34	3.97	±0.32	0.0041
Pre-HD creatinine (mg/dL)	10.76	±2.48	10.73	±2.54	10.78	±2.44	0.733
nPCR (g/kg/day)	1.28	±0.43	1.28	±0.43	1.28	±0.44	0.8284
TACurea (mg/dL)	41.87	±11.54	42.2	±12.3	41.55	±10.79	0.569
Potassium (meq/L)	4.93	±0.76	4.93	±0.77	4.94	±0.75	0.7899
Kt/V	1.51	±0.35	1.51	±0.42	1.5	±0.26	0.9566
URR	0.77	±0.06	0.76	±0.06	0.78	±0.06	<0.0001
Hematocrit (%)	31.88	±4.1	32.05	±4.26	31.72	±3.94	0.6287
Iron administration by vein							
No	463 (78.88)		236 (82.23)		227 (75.67)		0.0509
Yes	124 (21.12)		51 (17.77)		73 (24.33)		
Monthly EPO usage (1000U/month)*	16 (10,22)		16 (8,20)		16 (12, 22)		0.0979
Calcium (mg/dL)	9.76	±0.99	9.82	±1.01	9.71	±0.96	0.2305
Phosphate (mg/dL)	4.9	±1.49	4.89	±1.57	4.91	±1.42	0.8149
iPTH (ng/mL)*	181.5 (62.1, 455.2)		178.2 (53.4, 441.5)		188.1 (71.7, 478.25)		0.6106
Total cholesterol (mg/dL)	169.69	±36.21	168.94	±35.75	170.37	±36.66	0.6157
Glucose (mg/dL)*	96.0 (84.0, 128.0)		97 (85, 129)		94 (82, 127)		0.2219
Cardiothoracic ratio	0.49	±0.06	0.50	±0.07	0.48	±0.06	<0.0001

e group: e-intervention group; non-e group: non-e-intervention group; HD: hemodialysis; nPCR: normalized protein catabolic rate; TACurea: time-averaged concentration of urea; URR: urea reduction ratio; EPO: erythropoietin; iPTH: intact parathyroid hormone.

*The variables with skew distribution were presented as median (1st-quartile, 3rd-quartile) and tested with Mann–Whitney U-test accordingly.

^The p-value was calculated by multiple regression models while adjusted by age, gender, and HD duration.

## Discussion

Currently, the effects and potential benefits of e-intervention application in a health care system are a popular area of research. In 1977, Pollak *et al*. proposed that the objectives of adopting a new record system, such as an online computerized data handling system, to treat patients with renal diseases were: improving the decision-making processes, monitoring the quality of medical care, analyzing the data easily and rapidly, and serving as a useful new teaching model^10^. In the past two decades, many systematic reviews have reported the positive effects of e-interventions^10–12^. Chaudhry *et al*. reported that electronic information systems enhance the quality of medical care by increasing the compliance of medical guidelines, strengthening the monitoring of medical practices, and reducing medical errors^13^. Regarding to improving the quality of medical care, the main benefit of these systems is the decreased use of unnecessary medical resources. The main purpose of this study was to assess the effects of e-interventions on the quality of medical care and determine its potential benefits in patient data management in hospitals. Therefore, the two study groups were matched to improve comparability. Of the 1,208 patients in the database, a total of 932 eligible patients were included in the study after excluding cases with a HD duration < 1 year. The eligible patients were individually matched for sex, age, HD duration, diabetes mellitus (DM), and hypertension, and finally 600 matched patients were evaluated in this study. The Student *t* test and chi-squared test revealed that the 932 eligible patients and the final 600 matched patients did not exhibit significant differences in their age, sex, hypertension, DM, and HD duration (*p* > 0.05).

After applying electronic information technology to HD patient care, Pollak *et al*. observed that the mortality rate and hospital admission frequency of HD patients decreased considerably, particularly after the third year of system application^14^. In the current study, the e-intervention group had a higher 4-year survival rate (84.67%) than the non-e-intervention group (80.33%). The multivariate Cox regression analysis showed that the e-intervention was one of the determining factors for the 4-year all-cause mortality rate. Furthermore, our results demonstrated that the e-intervention reduced the annual number of hospitalization days at 1-year post e-intervention. Moreover, e-intervention systems facilitate patient medical care. Pollak *et al*. indicated that the system could assist physicians to adjust patients’ dry weight to prevent dialysis hypotension. The e-intervention systems enable rapid data review, thus enabling physicians and other health care professionals to perform adequate adjustments in medication prescriptions and medical orders^11^. Furthermore, the systems provide the data associated with anemia to physicians and help them in adjusting the erythropoietin dosage and providing timely prescription of iron supply^12^. In the present study, the e-intervention and non-e-intervention groups differed significantly in their 2-year all-cause mortality rates but not in their 4-year all-cause mortality rates. The e-intervention exerted short-term effects on outcomes during the first 2 years of application, demonstrating the multi-factorial nature of HD patients’ prognosis. We applied generalized estimating equation to examine the effects of time-dependent variables, including the albumin, hematocrit, and phosphate level, on the patient outcomes and observed that the non-e-intervention group had a higher mortality rate than the e-intervention group (HR = 3.039; 95% CI = 1.737~5.314). The e-intervention may exert independent effects beyond the pathways of time-dependent indicators on the mortality rates.

In addition to the e-intervention group, patients with relatively lower education level, unmarried, those with lower pre-HD creatinine levels, and those with higher sugar levels a higher 4-year all-cause mortality rate. Marriage was a protective factor for the 4-year all-cause mortality rate in this study patients. This finding is consistent with the findings of a previous study that applied the Social Adaptability Index in the dialysis population^15^. Regarding the co-morbidities, Lorch *et al*. demonstrated that co-morbid conditions are major determinants of outcomes^12^. Lower pre-HD serum creatinine levels associated with poor nutritional status have been evidenced as predictors of HD patients outcomes^16, 17^. In addition, high fasting glucose levels indicate poor sugar control in patients. The Choices for Healthy Outcomes in Caring for ESRD study showed that compared with arteriovenous fistula, the double lumen catheters were associated with increased degree of inflammation and higher mortality in incident HD patients^18^. However, this finding was not observed in our study. We analyzed the principal diagnoses for patient’s hospitalization during the first year of study and observed that infectious diseases (32.3%), cardiovascular or cerebrovascular diseases (21.3%), vascular access-associated disorders (18.3%), gastrointestinal disorders (15.9%), and others (12.2%) were associated with higher mortality rates in HD patients.

According to the data obtained before the e-intervention (Table 1) and the outcomes at 1-year post e-intervention (Table 3), the e-intervention group had a relatively higher URR, higher albumin levels, a lower cardiothoracic ratio^19^, and a higher HD blood flow rate than the non-e-intervention group. Studies have reported that vascular access type associated with the HD blood flow rate influences the mortality in HD patients^20, 21^. However, a Japanese study suggested that a low HD blood flow rate may benefit the survival rates in their population^22^. By contrast, the Dialysis Outcomes and Practice Patterns Study in Japan reported that patients with a lower HD blood flow rate (<180 mL/min) had a higher mortality rate than the patients in the reference group with a relatively higher HD blood flow rate (180–210 mL/min)^23^. Furthermore, Malaysian study showed that a higher HD blood flow rate is associated with a higher quality of life^24^. A high HD blood flow rate may be attributed to the effective maintenance of the vascular access. Moreover, adequate HD blood flow rates can lead to adequate clearance, which is demonstrated by an increased Kt/V and URR^23^. We performed Cox regression analysis for the 4-year all-cause mortality rates based on the biophysiological indicators measured 1-year post e-intervention. The HD blood flow rates were negatively associated with 4-year all-cause mortality rates (HR = 0.989, 95% CI = 0.984~0.993), but did not exhibit significant results in the multivariate-adjusted analysis. Therefore, additional studies are warranted to elucidate the causal relationship between the HD blood flow rate and mortality rate.

The present study has numerous limitations. First, this study has a retrospective design, and cases with missing medical data and records were excluded. Therefore, the number of cases was reduced, which might have affected the integrity of the study results. Second, we intended to analyze the effects of e-interventions, therefore, the two study groups were matched for sex, age, HD duration, DM, and hypertension and the complicated cases were excluded. Therefore, the mortality rate and hospitalization frequency of the study patients were lower than those of the other studies in Taiwan^25, 26^. The distribution of the causes of ESRD was also different compared with other studies in Taiwan^25^. Therefore, the predictors, such as serum phosphate and albumin levels, were closer to their normal ranges in the study patients and did not demonstrate a significant association with the mortality rates. Third, patients in the two study groups were from different hospital branches, and therefore some innate differences may have existed. Although the data were adjusted in the statistical analysis, interference effects cannot be completely ruled out. Fourth, the current health care system in Taiwan is a single-payer compulsory social insurance plan. Some prospective payment system policy interventions, including global budget, and pay-for-performance, were introduced during 2004–2005. Therefore, the data prior to 2005 were not included to avoid such historic effects on the results. Finally, because of the constant changes in the health care environment and health policies, the present results can only reflect the effects of the e-intervention during the specific study period and of the unique electronic information systems of the research institutes involved in the present study.

## Conclusion

Due to the rapid advances in medical technology and increasing attention on health and medical effectiveness, the objective of HD treatment has gradually changed from the passive replacement kidney functions to the active reduction of complications and improvement in discomfort during HD. HD is expected to not only prolong life but also improve the quality of life. The e-intervention adopted for HD care in the present study showed promising results. The e-intervention group had a significantly higher HD blood flow rate, fewer hospitalization days, and a relatively lower 4-year all-cause mortality rate than the non-e-intervention group.

The implementation of e-interventions is a current trend in medical management to simplify medical-related processes and save medical resources^27^. The use of an e-intervention to analyze and monitor medical processes can also help to provide safer medical services. However, e-interventions require substantial investments in hardware and software. In addition, medical personnel require education and training to appropriately operate these systems. Although e-interventions improved patient outcomes in the present study, additional studies are required to evaluate the cost effectiveness of such e-interventions.

## Methods

### Study patients and interventions

This retrospective was conducted using the clinical data abstracted from the Chang Gung Memorial Hospital (CGMH) information system. We evaluated the effectiveness of a quality improvement intervention by introducing an integrated information system for HD patient care. Although the analysis was performed using a prospective method, the nature and protocol of this study were reviewed and approved by the Institutional Review Board (IRB) of CGMH. The IRB provided an exemption certificate for this review (99–2617B). Therefore, written or verbal informed consent was not required from the study patients. All research methods in this study were performed in accordance with the approved guidelines.

**Figure 2:**
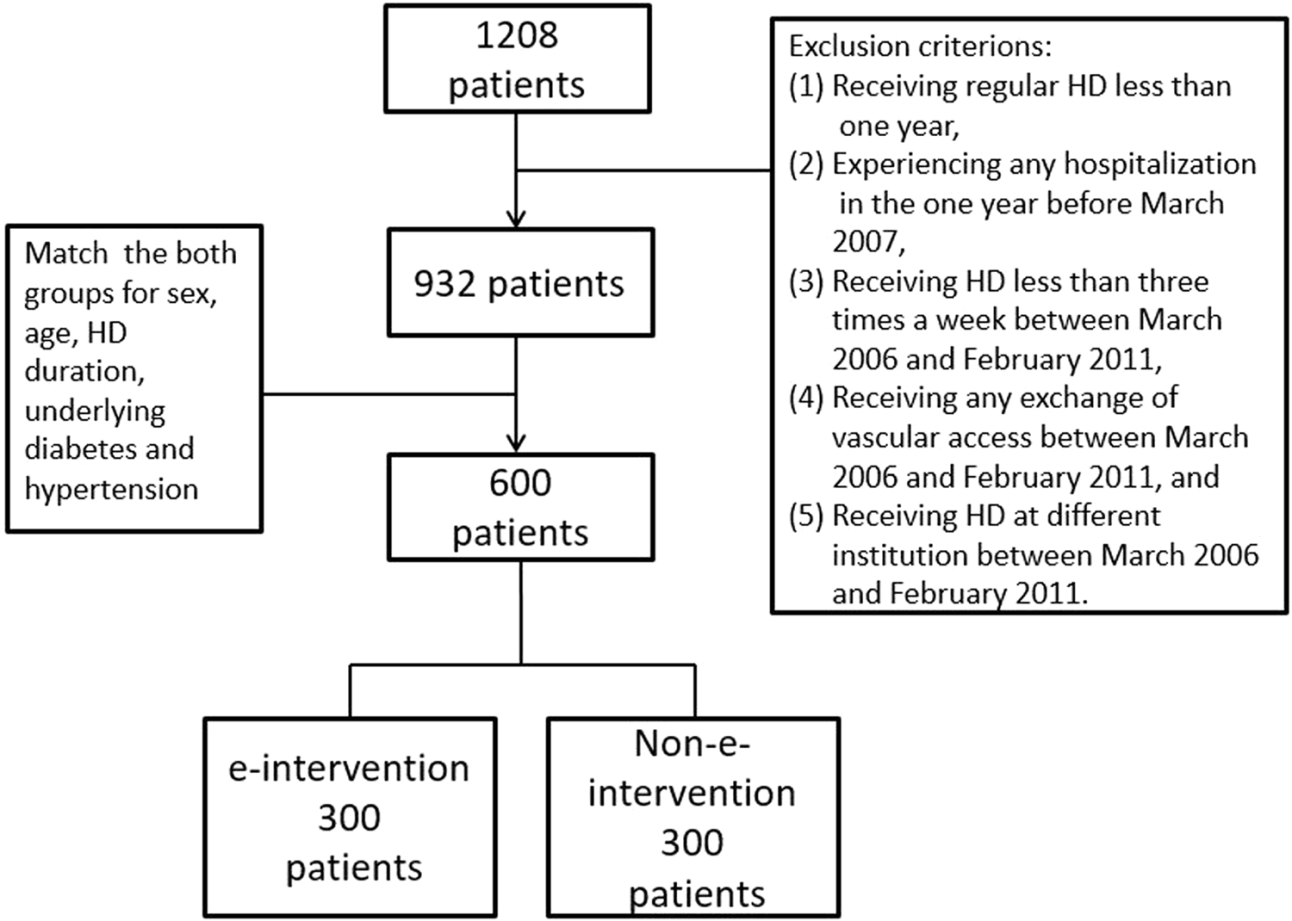
The consort diagram of study samples.

The patients who received regular HD at any of the three CGMHs (Taoyuan, Taipei, and Linkou) in March 2007 were included in this study. Of the 1,208 HD patients, 276 were excluded due to one of the following reasons: (1) receiving regular HD for < 1 year; (2) history of hospitalization within 1 year before March 2007; (3) receiving HD less than three times a week between March 2006 and February 2011; (4) receiving any alterations of the vascular access between March 2006 and February 2011; and (5) receiving HD at a different institution between March 2006 and February 2011. The remaining 932 HD patients were divided into the e-intervention group (patients who received HD at Taoyuan CGMH and were started on e-intervention from March 2007) and the non-e-intervention group (patients who received HD at Linkou CGMH and Taipei CGMH and did not receive e-intervention from March 2007) according to each patient’s medical care settings. After matching for sex, age ± 3 years, HD duration, DM and hypertension, a total of 600 HD patients (300 patients each in the e-intervention and non-e-intervention groups) were recruited. Figure 2 shows the consort diagram of the study patients.

**Figure 3:**
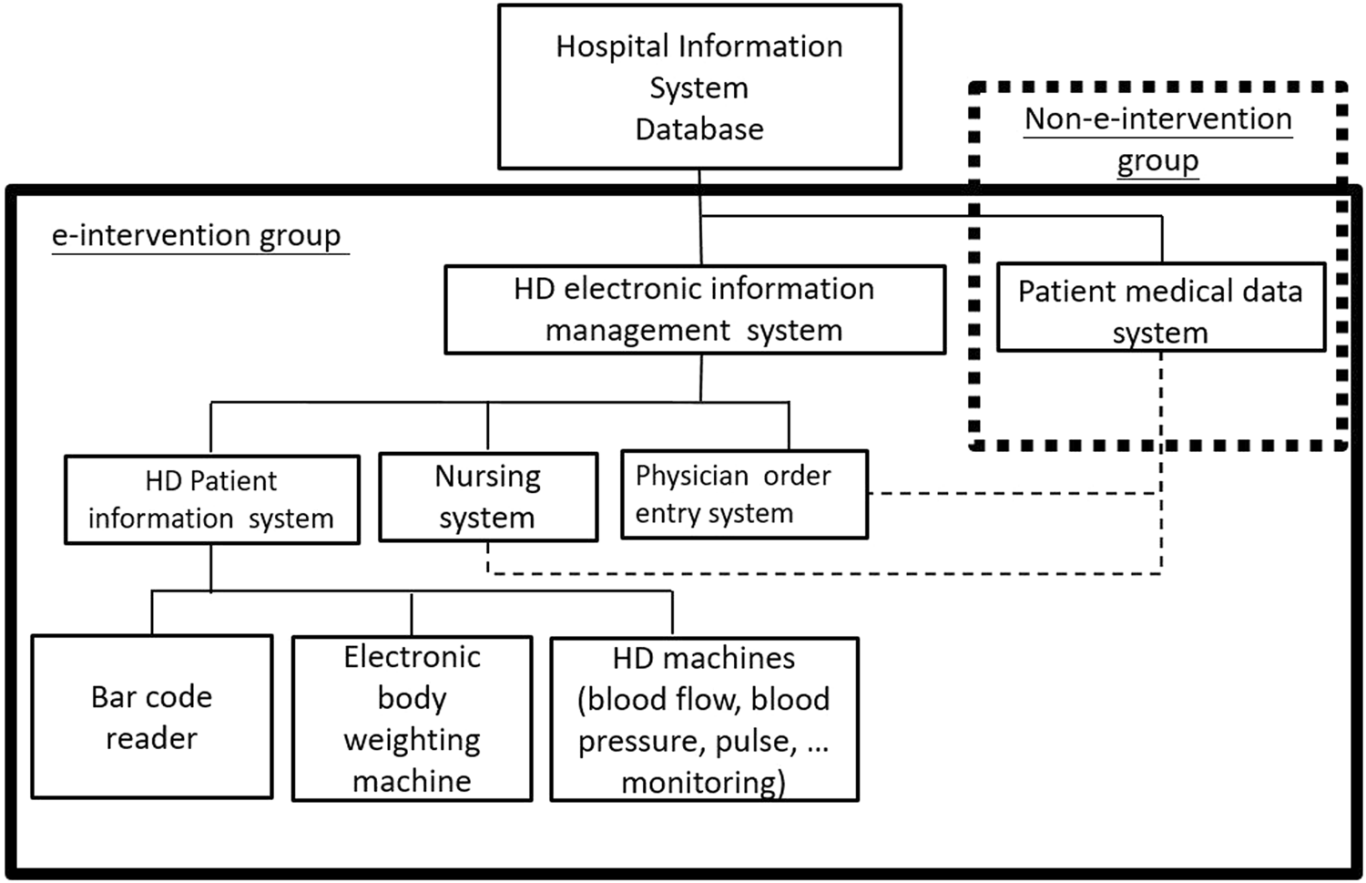
The architecture of the information systems utilized in this study.

The e-intervention involved the use of the HD electronic information management system of CGMH. It applied electronic information technology and integrated information from the HD patient information system, physician order entry system, and nursing system. It had access to the existing patient medical data system which provided medical records of all patients in CGMH. The e-intervention created a platform to share information with practitioners and patients and provide assisted patient-centered medical care. When a HD patient was admitted to the HD room, a code reader would record patient’s identity and bed number and link with results from an electronic body weighing machine. The pre-dialysis preparation, including dialyzer, dialysate, and heparinization preparations, was performed by a technician according to the data present in the electronic information system. After starting the HD process, the ultrafiltration volume setting was established on the basis of the dry weight and measured body weight data in the electronic information system. During HD, patient data including blood pressure, body temperature, blood flow rate, dialysate flow rate and vascular access pressure, were recorded automatically and uploaded into the electronic information system. The HD nurses were informed by an e-alert system if any abnormality was detected. The system also provided physicians’ orders to the HD nurses, who could promptly perform medical practices accordingly. Nearly twenty software interfaces in the electronic information system, including medical records, physicians’ orders, medication data, medical devices, medical materials, HD schedules, HD indicators, data statistics, pricing, administrative affairs, and staff’s duty scheduling, facilitated healthcare management. The major objectives of the e-intervention were to: (1) improve the efficiency of the medical care protocol by shortening the prescription time, prompting laboratory reports, and accelerating the acquisition of imaging information (electronic retrieval system for X ray images); (2) reduce the workload of medical personnel, including saving time on laboratory data retrieval, prompt prescription of medications on-line, and providing an expert assistance platform to assess the comprehensiveness of the patient data; (3) enhance the accuracy of medical processes including scheduling, prescriptions, laboratory reports, and medical records; and (4) increase patient safety and medical care quality through information integration. The patients in the e-intervention group were managed by the HD electronic information management system and the conventional patient medical data system. The patients in the non-e-intervention group were managed by their physicians and nurses using traditional paper-driven systems and conventional patient medical data systems. Figure 3 presents a detailed description of the e-intervention and non-e-intervention group.

### Data collection

**Figure 4:**
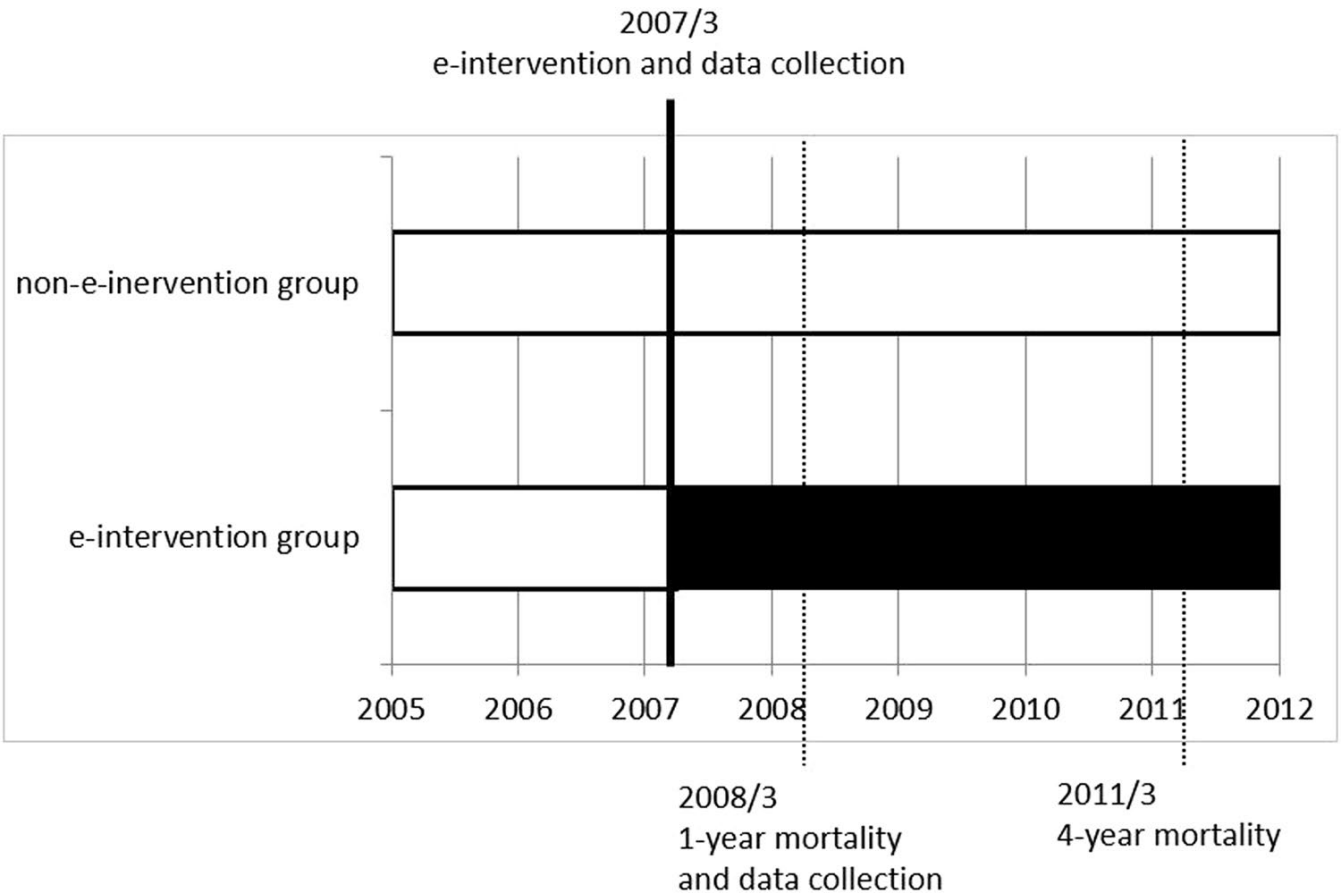
The time regimens of the study groups classified by e-intervention.

The basic demographic variables and clinical data, including age, sex, education levels, occupation, marital status, HD duration, causes of ESRD (including chronic glomerulonephritis, DM, hypertension, and other chronic conditions), and history of diseases and health conditions (including hepatitis B virus, hepatitis C virus (HCV), vascular access types, DM, hypertension, and body mass index^28^), of HD patients were assessed in March 2007 using a questionnaire and confirmed with a medical chart review. In addition, physiological and biochemical variables (including the daily amount of urine, dialyzer surface area, albumin, pre-HD creatinine, normalized protein catabolic rate^29^, time-averaged concentration of blood urea nitrogen^30^, potassium levels, Kt/V^31^, URR, haematocrit levels, monthly erythropoietin usage, calcium, phosphate levels, intact parathyroid hormone, total cholesterol, cardiothoracic ratio, and iron supply) before the e-intervention (in February 2007) and at 1-year post e-intervention (in March 2008) were obtained. Intravenous of iron administration was prescribed to patients according to the protocol of the CGMH HD room: hematocrit < 28%, transferrin saturation < 20% and ferritin level < 200 μg/mL, patients were intravenously administered with 1 g of Sucrofer^®^ (iron sucrose complex 2%, 20 mg Fe per mL, 5 mL per amp), divided to 10 times and was given one time a week for 10 weeks. During the 4-year follow-up period until February 2011, outcome variables, including total length of hospitalization and 4-year all-cause mortality rate were measured. Figure 4 presents a detailed description of the time study schedule.

### Statistical analysis

Numerical and categorical variables are presented as means ± standard deviations, and frequencies and percentages, respectively. The independent sample *t* test was used to compare the means of continuous variables and normally distributed data; otherwise, the Mann Whitney U test was used. The chi-squared test was used to compare the differences between categorical variables. A Cox regression model was used to determine the strength of the associations between the study groups and 4-year all-cause mortality, after examining (and adjusting for) the effects from other selected variables. A *p* value of <0.1 was pre-determined as the multivariable model selection criteria among variables with *p* < 0.05 in the univariate analysis after considering the effects of co-linearity among candidate variables. The results are expressed as multivariate-adjusted HR and corresponding 95% CIs. In stratified analysis, the causes of ESRD and the vascular access types were further categorized separately into groups and visualized using bar charts. Cumulative survival curves as a function of time were constructed using the Kaplan Meier approach, and the log rank test was conducted to determine their statistical significance. SAS software version 9.30 (Cary, NC, USA) was used for all statistical analyses in this study.

## References

[1] Wen CP (2008). All-cause mortality attributable to chronic kidney disease (CKD): a prospective cohort study based on 462,293 adults in Taiwan. Lancet..

[2] National Kidney Foundation. K/DOQI Clinical Practice Guidelines for Hemodialysis Adequacy. *Am J Kidney Dis*. **37**, S7–S64 (2001).

[3] De Palma JR (1971). Adequate hemodialysis schedule. N Engl J Med..

[4] Pollak VE (1990). Computerized medical information system enhances quality assurance. A 10-year experience in chronic maintenance hemodialysis patients. Nephron..

[5] Dean BB (2009). Review: use of electronic medical records for health outcomes research: a literature review. Med Care Res Rev..

[6] Vito D, Casagrande G, Bianchi C, Costantino ML (2015). How to extract clinically useful information from large amount of dialysis related stored data. Annual International Conference of the IEEE Engineering in Medicine & Biology Society..

[7] Alam A (2010). Computerization of hemodialysis records: a new era explored. Saudi Journal of Kidney Diseases & Transplantation..

[8] Devcic B, Jelic I, Racki S (2014). Management of hemodialysis patients using simple informatics program. Acta Medica Croatica..

[9] Wise ME, Lovell C (2013). Public health surveillance in the dialysis setting: opportunities and challenges for using electronic health records. Seminars in Dialysis..

[10] Pollak VE, Buncher R, Donovan ER (1977). On-line computerized data handling system for treating patients with renal disease. Arch Intern Med..

[11] Pollak VE (1983). Computerization of the medical records: Utility in the care of patients with end-stage renal disease. Kidney Int..

[12] Lorch, J. A., Pollak, V. E. Computerized Patient Record in Dialysis Practice. In: Replacement of Renal Function by Dialysis, 5th ed. England Kluwer Academic Publishers, *London. pp*. 2004, 539–553 (2004).

[13] Chaudhry B (2006). Systemic Review: Impact of health information technology on quality, efficiency, and costs of medical care. Ann Intern Med..

[14] Pollak, V. E., Lorch, J. A. Effect of electronic patient record use on mortality in End Stage Renal Disease, a model chronic disease: retrospective analysis of 9 years of prospectively collected data. *BMC Med Inf Decis Mak*. **7**, page number not for citation purposes (2007). 10.1186/1472-6947-7-38 PMC2238736 18045495

[15] Sandhu GS (2011). Social Adaptability Index: application and outcomes in a dialysis population. Nephrol Dial Transplant..

[16] Chua HR (2014). Predicting first-year mortality in incident dialysis patients with end-stage renal disease - the UREA5 study. Blood Purification..

[17] Kim CH (2014). LDL cholesterol affects clinical outcomes in incident hemodialysis patients during the early stages of dialysis. Blood Purification..

[18] Banerjee T (2014). Vascular access type, inflammatory markers, and mortality in incident hemodialysis patients: the Choices for Healthy Outcomes in Caring for End-Stage Renal Disease (CHOICE) Study. American Journal of Kidney Diseases..

[19] Chen KH (2011). Cardiothoracic ratio association with mortality in patients on maintenance peritoneal dialysis. Therapeutic Apheresis & Dialysis..

[20] Dhingra RK, Young EW, Hulbert-Shearon TE, Leavey SF, Port FK (2001). Type of vascular access and mortality in US hemodialysis patients. Kidney Int..

[21] Canaud B (2008). DOPPS estimate of patient life years attributable to modifiable hemodialysis practices in France. Nephrol Ther..

[22] Pison RL (2009). Facility Hemodialysis Vascular Access Use and Mortality in Countries Participating in DOPPS: An Instrumental Variable Analysis. Am J Kidney Dis..

[23] Kimata N (2014). Gender, low Kt/V, and mortality in Japanese hemodialysis patients: Opportunities for improvement through modifiable practices. Hemodial Int..

[24] Nor Baizura MY, Chan YM, Zalilah Mohd S, Choo BH (2013). Factors Associated with Quality of Life among Hemodialysis Patients in Malaysia. PLOS ONE..

[25] Hwang SJ (2010). Impact of the clinical conditions at dialysis initiation on mortality in incident haemodialysis patients: a national cohort study in Taiwan. Nephrol Dial Transplant..

[26] Chang YK (2012). A Comparative Assessment of Survival Between Propensity Score-Matched Patients With Peritoneal Dialysis and Hemodialysis in Taiwan. Medicine..

[27] Hayrinen K, Saranto K, Nykanen P (2008). Definition, structure, content, use and impacts of electronic health records: A review of the research literature. Int J Med Inf..

[28] Kopple JD, Zhu X, Lew NL, Lowrie EG (1999). Body weight-for-height relationships predict mortality in maintenance hemodialysis patients. Kidney Int..

[29] Leavey SF, Strawderman RL, Jones CA, Port FK, Held PJ (1998). Simple nutritional indicators as independent predictors of mortality in hemodialysis patients. Am J Kidney Dis..

[30] Lowrie EG, Laird NM, Parker TF, Sargent JA (1981). Effect of the Hemodialysis Prescription on Patient Morbidity — Report from the National Cooperative Dialysis Study. N Engl J Med..

[31] The National Health Insurance Statistics. National Health Insurance Administration, Ministry of Health and Welfare, Taiwan. Available: http://www.nhi.gov.tw/English/webdata/webdata.aspx?menu=11&menu_id=296&WD_ID=296&webdata_id=4229 (2011).

